# Male and LGBT survivors of sexual violence in conflict situations: a realist review of health interventions in low-and middle-income countries

**DOI:** 10.1186/s13031-020-0254-5

**Published:** 2020-02-26

**Authors:** Ligia Kiss, Meaghen Quinlan-Davidson, Laura Pasquero, Patricia Ollé Tejero, Charu Hogg, Joachim Theis, Andrew Park, Cathy Zimmerman, Mazeda Hossain

**Affiliations:** 1 Institute for Global Health, University College London, London, UK; 2 All Survivors Project, Vaduz, Liechtenstein; 3 London School of Economics, London, UK; 4 Independent Researchers, Tokyo, Japan; 5 Independent Researchers, Los Angeles, USA; 6 Department of Global Health and Development, London School of Hygiene and Tropical Medicine, London, UK; 7 Centre for Women, Peace and Security, London School of Economics, London, UK

**Keywords:** Conflict-related sexual violence, Men, boys, and LGBT survivors, Medical interventions, Mental health and psychosocial support interventions, Systematic realist review, Realist synthesis

## Abstract

Conflict-related sexual violence (CRSV) against women and girls has been the subject of increasing research and scholarship. Less is known about the health of men, boys and lesbian, gay, bisexual, transgender (LGBT) and other gender non-binary persons who survive CRSV. This paper is the first systematic realist review on medical, mental health and psychosocial support (MHPSS) interventions that focusses on male and LGBT survivors of CRSV. The review explores the gender differences in context, mechanisms and outcomes that underpin interventions addressing the health and psychosocial wellbeing of male and LGBT survivors. The aim is to contribute to the design and delivery of gender-sensitive and, when needed, gender-specific approaches for interventions that respond to specific needs of different groups of all survivors. We conducted a systematic search of academic and grey literature to identify medical and MHPSS interventions that included men, boys and LGBT survivors. We identified interventions specifically targeting women and girls that we used as comparators. We then purposively sampled studies from the fields of gender and health, and sexual abuse against men and LGBT people for theory building and testing. We identified 26 evaluations of interventions for survivors of CRSV. Nine studies included male survivors, twelve studies focussed exclusively on female survivors and one study targeted children and adolescents. No intervention evaluation focussed on LGBT survivors of CRSV. The interventions that included male survivors did not describe specific components for this population. Results of intervention evaluations that included male survivors were not disaggregated by gender, and some studies did not report the gender composition. Although some mental health and psychosocial consequences of sexual violence against men and boys may be similar among male and female survivors, the way each process trauma, display symptoms, seek help, adhere to treatment and improve their mental health differ by gender. Initiatives targeting male and LGBT survivors of CRSV need to be designed to actively address specific gender differences in access, adherence and response to MHPSS interventions. Models of care that are gender-sensitive and integrated to local resources are promising avenues to promote the health of male and LGBT survivors of CRSV.

## Background

Over the past two decades, sexual and gender-based violence against women and girls in conflict situations has received increasing attention [1], leading to a marked progress in research and the development of interventions to identify survivors and support their health and protection needs. At the same time, however, the health of men, boys, and lesbian, gay, bisexual, transgender (LGBT) and other non-binary people exposed to sexual violence in conflict has been insufficiently addressed by research and the UN policy agenda [2–9]. This article uses the umbrella term LGBT to include a number of groups defined by diverse sexual orientations and gender identities. We defined gender as socially constructed attributes, behaviours, roles, and norms associated with each sex [10]; and gender identity as an individual’s felt sense and experience of their own gender [11]. In addition to men and boys, our review focussed on sexual minority men and transgender people, though none of studies identified presented data disaggregated by the categories within the LGBT notion. Therefore, our review does not provide a basis for conclusions about each of these groups individually. Most articles identified in the review referred to LGBT, sometimes including intersex persons in the definition of sexual and gender minorities. In this paper, we use the term LGBT to refer to lesbian, gay, bisexual, transgender (LGBT) and other non-binary persons.

Sexual violence against men and boys has often been recognised as torture, mutilation or degrading treatment [8, 9, 12], omitting the gendered and sexual aspects of these abuses [6, 13]. Acts of sexual violence against men and boys include anal and oral rape and other forms of victimisation, including gang rape, enforced sterilisation, mutilation, castration, blunt trauma to genitals, forced nudity, forced masturbation, forced rape perpetration, and forced witness to sexual violence against family members or peers [2, 3, 12, 14, 15]. This sexual violence can occur in many settings, including detention centres, military sites, refugee camps and people’s homes during and after conflict [16, 17].

Sexual violence in conflict can be used as a form of torture aiming to inflict psychological suffering, terrorise, humiliate, disempower and break down the identity of perceived enemies or political prisoners [2, 14]. Perpetrators of sexual violence against men often seek to impose domination, power and control through their acts [13, 18]. Prevailing gender norms that manifest in sexual violence against men and boys also appear in sexual violence against women and girls [8].

Sexual abuse has been used for torture and interrogation, for initiation into military or paramilitary forces, to destabilise families, terrorise communities, hinder social cohesion, and to perpetrate ethnic cleansing [15]. Impunity for perpetrators is usually the norm [8]. Sexual abuses often occur jointly with other crimes, such as killing, looting, pillage, forced displacement and arbitrary detention [1]. Research documenting the prevalence of CRSV against men is extremely limited, but studies indicate that the phenomenon is widespread. For example, a cross-sectional population survey in Liberia found that 32.6% of male former combatants experienced sexual violence [19]. Another population survey in DRC estimated the prevalence CRSV among men at 23.6% [20]. Research from Sri Lanka estimates that 9–21% of men experienced some form of CRSV [21]. On the other hand, a cross sectional survey In Cote d’Ivoire found that less than 1% of men in conflict-affected communities reported sexual violence from a combatant or other official [22].

Violence against LGBT people in conflict settings has been recognised by the United Nations as a form of gender-based violence (GBV) that is often motivated by homophobic and transphobic attitudes and directed at those perceived as defying hegemonic gender norms [23]. In post-conflict settings, LGBT people often experience harassment and need to hide their sexual orientation or gender identity. Abuse and violence by security agents, local community members and other asylum seekers or refugees is common. Additionally, ‘honour killings’ may target LGBT individuals [24]. Exclusion from economic opportunities or from access to services may also occur as a result of homophobic attitudes [7].

The mental health consequences of sexual violence can be severe and long-lasting. The list of symptoms and antisocial behaviour associated with sexual torture, trauma and violence includes: impaired memory and concentration, low self-esteem, difficulty relating to others, difficulty engaging in intimate relationships, anger outbursts, explosive rage, emotional withdrawal, detachment, lack of adherence to family life, self-mutilation, suicidal behaviour, sleep disturbances, nightmares, apathy, helplessness and cognitive impairment. Alcohol and drug abuse are also reportedly common among survivors [2, 3, 5, 18, 25, 26]. Additionally, male survivors of sexual violence may be particularly concerned about threats to their perceived notions of masculinity, self-doubt about their sexual orientation, fear of rejection, and concerns about not being able to prevent the abuse, and about re-victimisation [27, 28]. Access to care for male victims can also be challenging, as they are less often identified by health providers as being in need of protection and psychosocial assistance than female survivors [9].

Physical health consequences of sexual violence against men and boys include sexually-transmitted infections (STI), HIV, infertility, sexual dysfunctions, impotence, genital infections, genital injuries, blood in stools, abscesses and rupture of the rectum, diarrhoea, loss of body parts, chronic pain, palpitations and headaches [2, 25, 29–31]. Non-genital and rectal injuries may include bruises and contusions, lacerations, ligature marks to ankles, wrists and neck and pattern injuries (hand prints, finger marks, belt marks, bite marks) [31].

Sexual violence against males, as other forms of torture, affects not only the survivors, but also their families and communities. Many survivors are often abandoned or rejected by their families because of the stigma surrounding sexual violence against men [2, 32]. Increased perpetration of violence, substance abuse and self-imposed isolation from the family and community can also increase male isolation and disrupt family life in the aftermath of male sexual abuse [31]. For those families, the loss of a working-age male can seriously affect their livelihood options [2]. Some survivors are isolated and ostracised by their community [33], which poses additional challenges for their recovery and economic survival [32].

LGBT persons who survive sexual violence may be confronted with the additional challenge of a heightened sense of vulnerability linked to their sexual orientation or gender identity. This type of hate crime may also instil fear and pressure among LGBT individuals to hide their sexual orientation or gender identity as a means to protect themselves from violence, and thus further aggravate mental health symptoms [29].

Despite the severe health and social burden associated with CRSV, virtually no evidence exists on how medical, mental health and psychosocial support (MHPSS) interventions work for men, boys and LGBT survivors of CRSV. One realist review was conducted on female CRSV [34], and two systematic reviews on CRSV interventions have been carried out [35, 36]. However, male and LGBT survivors were either not explicitly considered, or due to the lack of specific quantitative studies on these groups, did not allow for inclusion in the systematic reviews. This paper builds on this body of knowledge to examine the mechanisms through which current medical and MHPSS interventions may work (or not) for men, boys and LGBT survivors of CRSV and under which circumstances.

## Methods

This study aimed to identify how, why, and in what circumstances existing medical and MHPSS interventions improve physical and mental health outcomes among male and LGBT survivors of CRSV. We chose to conduct a realist review as it aims to identify how, why, and what programmes or interventions work in particular settings and contexts. It involves trying to determine causal relationships between outcomes, underlying mechanisms, and contexts [37]. Indeed, realist analyses are structured using context-mechanism-outcome (CMO) configurations, in which findings on context and mechanisms are used to explain how interventions produce determined outcomes among diverse subgroups in exposed populations. We followed the RAMSES quality standard for realist reviews [38]. The protocol for the systematic realist review is registered in PROSPERO (reference: CRD42019135072).

The review comprised four stages, as described below.

### Stage one

In the first stage, we conducted a literature review, including a rapid assessment of the literature informed by experts, recent systematic and narrative reviews of medical and MHPSS interventions for male and LGBT survivors of CRSV. We systematically searched the following electronic bibliographic databases: Pubmed, EMBASE, MEDLINE, PsycInfo, and Web of Science. Articles that focussed on medical, mental health, or psychosocial interventions and targeted men, boys, and adolescents in various humanitarian or conflict settings in low-and-middle-income countries met inclusion criteria. High-income settings were excluded so that we could learn from interventions undertaken in low-resource settings. Electronic searches were complemented by reference list screening, citation tracking of included materials in Web of Science and Google Scholar, hand searches of relevant websites, including the United Nations High Commissioner for Refugees (UNHCR), United Nations Population Fund (UNFPA), United Nations Children’s Fund (UNICEF), World Health Organization (WHO), International Rescue Committee (IRC), International Organization for Migration (IOM) and Médecins Sans Frontières (MSF). Expert recommendations were also included. We did not specifically search for sexual and reproductive health interventions in our review, although this was not an exclusion criterion for the review Table 1.

**Table 1:** Search terms for the realist review

Type of Intervention	Intervention words	Targeted group	Type of sexual violence	Setting	Location
Medical OR MHPSS OR mental health OR psychosocial OR psychological OR psychiatric	Intervention OR Initiative OR project OR program OR services	Male OR men OR boy OR adolescent OR LGBTI OR transgender OR homosexual men OR children	Sexual violence OR SV OR sexual trauma OR sexual torture OR sexual abuse OR sexual exploitation and abuse (SEA)	Conflict settings OR humanitarian settings OR emergency settings OR emergencies OR armed conflict OR conflict sites OR war zones OR displacement sites OR refugee settings OR refugee camps	Low and middle income countries OR LMIC

Following procedures from previous systematic reviews [36], we also searched ALNAP, a consortium of academics, UN agencies, donors, international and national NGOs, representatives from the Red Cross/Crescent Movement, and consultants that facilitates learning about how to improve humanitarian crises responses (ALNAP). The key term for this search was sexual violence, complemented with the following tags: assessment & analysis; conflict, violence & peace; evaluation-related; impact assessment; joint evaluations; evidence; feedback mechanisms; health; psychosocial; monitoring; and current learning and evaluation.

In Stage One, we systematically identified and extracted evidence on medical and MHPSS interventions for male, female and LGBT persons who self-identified or are identified by researchers, statutory or voluntary agencies as having experienced CRSV. Study participants included survivors of CRSV or other stakeholders (e.g. professionals involved in providing the intervention). No age restriction was applied.

We included medical and MHPSS interventions delivered by public, private, or charitable organisations to men, boys, and LGBT persons who experienced sexual violence in conflict-affected settings only, regardless of intervention (e.g. healthcare, community-based).

MHPSS interventions were defined as any non-pharmacological or biological interventions, activity or strategy delivered with the intention of improving mental health, functioning, or wellbeing (including social aspects such as social support), whether as primary or secondary outcomes. Interventions included could have been provided on an individual or group basis, or at the community level (e.g. awareness raising). They could also have been provided by various types of workers or agents; and could be primarily psychological (e.g. cognitive based therapy) or social (e.g. livelihoods, legal support, accommodation) [39]. It was anticipated that the intervention models may be highly divergent. Medical interventions were defined as any interventions that delivered medical services to treat or prevent immediate and potential long-term consequences of sexual violence, including STIs prevention and treatment, HIV prevention, pregnancy prevention, and vaccine-preventable diseases (tetanus, HepB and C) [31].

We excluded studies and materials that did not assess or evaluate (quantitatively or qualitatively) medical or MHPSS interventions related to sexual violence in conflict settings. We also excluded studies reporting the results of pharmacological interventions. Studies that did not explicitly discuss or provide evidence for the link between the intervention and outcome, and/or present methods that would enable links to be identified, were also excluded.

We extracted the evidence into a series of matrices using a pre-piloted extraction form in MS Excel and included the following information: type of intervention, intervention activities, context, resources, mode of delivery, mechanisms of change, outcome measures, and results. We stratified the studies by gender, age group, and intervention level. Studies on women and girls were used as comparators.

### Stage two

The second phase of our review consisted of definitions and theory development. Resources identified in Stage 1 were examined for intermediate and primary outcomes, initial mechanisms, mid-range theories, and patterns that linked the outcomes with intervention characteristics and contexts, suggesting potential mechanisms of change. Outcomes from Stages 1 and 2 were discussed with a panel of experts in the field during a workshop with members of the Research Advisory Group and key international stakeholders. The experts included representatives from the United Nations Population Fund (UNFPA) at the headquarter and country (Turkey) levels; World Health Organisation (WHO); International Rescue Committee (IRC); International Organisation for Migration (IOM) in the Central African Republic; the United Nations High Commissioner for Refugees (UNHCR); The Havens, Kings College Hospital NHS Foundation Trust; and Médecins Sans Frontières. The expert input was used to refine intervention theories and the Context-Mechanism-Outcome (CMO) configurations. Based on expert feedback, we designed Stage Four, a review of guidelines (described below).

The middle-range theories (i.e. theories that are limited in scope describing specific phenomena, vs “grand” social theories) resulting from this process provided the basis for the formulation of search strategies in Stage Three.

### Stage three

In Stage 3, we conducted a further review of the literature to develop and refine the middle range theories developed in Stages 1 and 2 (sexual violence survivors). The search strategy was developed on the basis of the preliminary findings on the mechanisms identified in Stage 2, such as results on service use by male survivors and provider’s awareness about male and LGBT persons’ experiences of CRSV. We used a purposive sampling strategy to address specific questions for theory building and testing, as identified in the previous review phases and following the realist review methods proposed by Pawson and colleagues [37] and further described by Croft-Malone and colleagues [40].

Since most of the evidence identified in our review was based on studies with women, or in which results were not disaggregated by gender, the main objective of this phase was to explore the applicability of intervention theories and generalisability of findings to male and LGBT survivors of CRSV. Based on results from the first review stages, in this third stage, the searches focussed on gendered aspects of access to health services, disclosure of sexual violence, acceptance and adherence to MHPSS interventions, and barriers to care. We searched the literature on gender and health, and on male experiences of sexual abuse during childhood and military services for theoretical insights. We stopped searches when we agreed we reached the point of saturation, as recommended by Croft-Malone and colleagues [40].

No restrictions were placed on publication format: materials were eligible for inclusion if they were, for example, published as peer-reviewed journal articles, conference proceedings, theses and dissertations, books, and reports. We prioritised the inclusion of systematic or realist reviews when available, proceeding to reference search for an overview of the evidence.

### Stage four

The consultation with experts and key international stakeholders (hereby experts) resulted in a stage Four of the review. This stage aimed at assessing to what extent and in what contexts well-known international UN and inter-agency literature for practitioners and policy experts – including guidelines, protocols, manuals and other documentation (hereby referred to generally as “guidelines”)– acknowledges male and LGBT survivors of sexual violence and provides specific guidance on service provision for these groups. Following expert advice, Stage Four included a rapid review of thirty-eight international guidelines providing guidance on medical and MHPSS responses for survivors of sexual violence including in conflict settings. The guideline review aimed to identify mechanisms and approaches explicitly or specifically addressing men, boys and LGBT survivors, independent of process or outcome evaluations. This additional stage was undertaken with the experts’ justification that the CRSV field does not have a strong tradition in robust intervention evaluations, and, therefore, there was a need to recognise recommendations that were drawn from policy-makers’ and providers’ assessments of the evidence, and their clinical and expert experiences.

Guidelines evaluated under the rapid review were selected based on experts’ recommendations and searches of relevant websites, including the World Health Organization (WHO), Global Protection Cluster (GPC) GBV and CP AoR, United Nations High Commissioner for Refugees (UNHCR), United Nations Population Fund (UNFPA), United Nations Children’s Fund (UNICEF), International Rescue Committee (IRC) and Inter-Agency Working Group on Reproductive Health in Crises (IAWG) among others. Inclusion criteria were: guidelines authored or endorsed by the UN and inter-agency coordination bodies which are (a) medical and MHPSS guidelines which include or address sexual and gender-based violence (to any extent); and (b) guidelines in other sectors, such as GBV or Child Protection which mention components of response linked to the health and/or MHPSS sectors. Where different editions exist for several guidelines, the successive editions of the same guidelines were analysed in order to assess change from one edition to the next one(s). The list does not aim to be exhaustive. Only UN and inter-agency bodies publicly-available guidelines were taken into account and therefore neither global reports, or regional and national guidelines, or published guidelines by international and national non-governmental organisations (NGOs/INGOs), or internal/unpublished organisational or other guidelines were included. At the time of writing, the 2019 WHO newly-revised Clinical Management of Rape (CMR) and Intimate Partner Violence Survivors guidelines are not yet publicly distributed and were therefore not included.

We first assessed, whether each guideline acknowledged and/or mentioned men and boys and LGBT among potential survivors of sexual violence. Then, for those guidelines which acknowledge male victimisation, we proceeded to analyse in what context(s) male and LGBT survivors are acknowledged, identifying whether specific needs, risks and vulnerabilities are taken into account and analysed and what type of guidance is provided on how to address these risks and needs. Additionally, we assessed to what extent guidelines incorporated an intersectional lens; in particular, we looked at how age, (dis)ability, health status, economic status, displacement status and other factors of potential diversity / vulnerability / power differentials of survivors were taken into consideration in guidance provision. Finally, for guidelines that were not first editions, we tried to assess any change / progress from one edition to the next one(s).

## Results

### The evidence on interventions targeting male and LGBT survivors of CRSV

#### Evidence-base

**Fig. 1:**
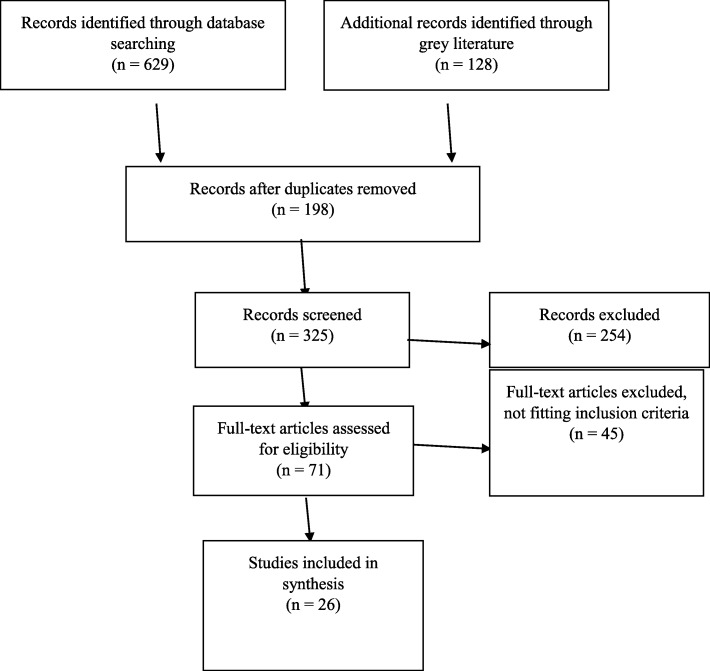
Flow diagram: number of articles selected in each stage of the search strategy

A total of 629 articles were initially retrieved in the academic database search, of which 431 articles were duplicates and discarded. Titles and abstracts were reviewed for 198 articles in the academic search, of which no studies met inclusion criteria. Additional records identified through reference list screening and citation tracking of included materials on Web of Science and Google Scholar yielded 124 articles, of which 22 articles met inclusion criteria. Grey literature searching of UNHCR, UNFPA, UNICEF, WHO, IRC, IOM, and MSF websites yielded a total of 4 articles (Fig. 1).

Table 2 describes the studies included in this review. Note that in the table, the studies identified as “male inclusive” indicate interventions that included both men and women. The studies that are labelled as “female specific” are interventions exclusively focussed on women.

**Table 2:** Selected studies for review: Evaluations of interventions targeting survivors of sexual violence

Study	Country	Intervention	Target Group	Intervention Components	Study Design
Bennett et al. (2017) [41]	Democratic Republic of Congo (DRC)	Medical, psychological, legal, socioeconomic	Male inclusive	Community sensitisation, medical care, legal services, psychosocial services, and income-generating activities	Implementation, description data
Bolton et al. (2014a) [42]	Iraq (KRI)	Psychological	Male inclusive	12 session Brief Behavioural Activation Treatment for Depression (BATD), 12 sessions of cognitive processing therapy (CPT), and waitlist control	Randomised Controlled Trial (RCT)
Bass et al. (2016) [43]	Iraq (KRI)	Psychosocial	Male inclusive	Healthcare provider capacity building, 6–12 sessions of trauma-informed treatment	RCT
Weiss et al. (2015) [44]	Iraq (KRI)	Psychological	Male inclusive	8–12 weekly individual sessions of Common Elements Treatment Approach (CETA)	RCT
Roka et al. (2014) [45]	DRC	Medical, psychological	Male inclusive	Community sensitisation, comprehensive medical and psychological care	Retrospective cohort study
Mooren et al. (2003) [46]	Bosnia	Psychological	Male inclusive	Community sensitisation and brief-trauma focused therapy	Baseline, follow-up survey of cases in the system
Wagner et al. (2012) [47]	Iraq (KRI)	Psychological	Male inclusive	Internet-based cognitive behavioural therapy over 5 week period	Pilot study, baseline and follow-up survey
Bolton et al. (2014b) [48]	Thailand (Burmese refugees)	Psychological	Male inclusive	CETA 7–13 weekly sessions	RCT
Bernath (2013) [49]	Rwanda	Psychosocial, medical, police and legal services	Male inclusive	Medical care provision, community sensitisation, psychological services	Qualitative interviews, implementation/description data
Kohli et al. (2012) [50]	DRC	Medical and psychological services	Female specific	Community sensitisation, medical services	Implementation, description data
Bass et al. (2013) [51]	DRC	Psychological	Female specific	Cognitive Processing Therapy (CPT) (11 group sessions) versus individual support	RCT
Hustache et al. (2009) [52]	Republic of the Congo	Psychological	Female specific	Counselling sessions (1–4 sessions)	
Lekskes et al. (2007) [53]	Liberia	Psychosocial	Female specific	Individual and group counselling (8 sessions)	Pre- and post-test, qualitative interviews
Doucet et al. (2012) [54]	Sierra Leone	Psychosocial	Female specific	Social work counselling program	Qualitative interviews
Allon et al. (2015) [55]	DRC	EMDR therapy	Female specific	Individual therapy + 2 sessions of standard Eye Movement Desensitisation and Reprocessing (EMDR) therapy versus group therapy + EMDR- Integrative Group Treatment Protocol (IGTP)	Pre- and post-survey
Hall et al. (2014) [56]	DRC	Psychological	Female specific	CPT (1 individual session and 11 weekly group sessions) versus individual support	RCT
PHD (2012) [57]	Nepal	Medical, psychosocial, legal, livelihood, shelter, and referral services	Female specific	Mobile health camp that referred clients and survivors to psychosocial and legal support, shelter, rehabilitation, and medical surgeries	Survey, qualitative interviews
Kohli et al. (2013) [58]	DRC	Psychosocial	Female specific	Family mediation	Qualitative interviews
Walstrom et al. (2013) [59]	Rwanda	Psychosocial	Female specific	Trauma counselling and support groups to HIV positive women	Qualitative interviews
Manneschmidt et al. (2009) [60]	Afghanistan	Psychosocial	Female specific	Counselling programme	
Schulz et al. (2006) [61]	Bosnia	Psychosocial	Female specific	CBT	Case study
O’Callaghan et al. (2013) [62]	DRC	Psychological	Girls	Trauma-focused CBT	RCT
Mbeya et al. (2018) [63]	CAR	Psychosocial	Service	Healthcare provider capacity building multimedia tools	
Tanabe et al. (2013) [64]	Burma	Medical and basic psychosocial care	Service	Healthcare provider capacity building	Qualitative
Smith et al. (2013) [65]	Refugee camps in Kenya and Ethiopia	Medical	Service	Multi-media training tool to train healthcare providers on clinical care of CRSV	Pre- and post-test survey, in-depth interviews, medical record audits
IRC (2012) [66]	Refugee camps in Ethiopia and Kenya, post-conflict in DRC, and urban settings in Jordan	Medical care	Service	Training program to improve the clinical care of sexual assault survivors using multimedia training tool	Survey, qualitative interviews, medical record audit

Sixteen studies were conducted in Africa (Democratic Republic of Congo (DRC), Ethiopia, Kenya, Rwanda, Republic of the Congo, Liberia, Sierra Leone, Burundi, and Central African Republic (CAR)), four studies in the Middle East (Iraq and Jordan), four studies in Asia (Thailand, Nepal, Afghanistan, and Burma), and two in Eastern Europe (Bosnia). Nine studies focussed on interventions for survivors of CRSV, including male survivors. Twelve studies focussed exclusively on female survivors, four studies targeted service provision, and one study targeted children and adolescents.

Results of evaluations that included both male and female survivors were not disaggregated by gender, and some studies did not report the gender composition of the research population. For studies that did provide the gender composition (men and women), the level of male participation ranged from 13% [47] to 68% [44]. None of the studies explicitly targeted the LGBT population nor did any of the studies seek to identify the sexual orientation or gender identity of participants. Interventions that included male participants consisted of 3 multi-sectoral packages (2 in the DRC and 1 in Rwanda) [41, 42, 45, 49], and 6 psychological treatments (4 in Iraq, 1 in Bosnia and 1 among Burmese survivors in Thailand) [43, 44, 46, 47, 51]. Another 12 studies were carried out exclusively with women, although a health education component of one study did include all members of the community [50]. Interventions that included only women were: two multisectoral packages (1 in DRC, 1 in Nepal) [50, 57]; and psychological interventions (4 in DRC, 1 in Rwanda, 1 in Republic of the Congo, 1 in Liberia, 1 in Sierra Leone; 1 in Afghanistan; 1 in Bosnia) [50–54, 56, 58, 60, 61]. One study was a trauma-informed CBT intervention for girls [62]. A total of 4 studies focussed on interventions to improve health services by training healthcare providers in medical services (in Kenya, Ethiopia, CAR, post-conflict DRC, urban settings in Jordan, and Burma) [63, 64].

Evaluation methods in the studies included in the reviews ranged from RCTs to qualitative interviews and medical records audit.

#### Effectiveness of MHPSS interventions

Findings from evaluations of MHPSS interventions that included men and boys reported effectiveness in reducing symptoms of depression, anxiety, PTSD, dysfunction or post-traumatic grief [42–44, 48]. No data on effect-size by gender were published in these evaluations. Therefore, we do not know whether the interventions were equally effective for women and men, or whether they were effective at all among male survivors (Table 3). Evaluations also presented limited information on service outreach, which restricts the conclusions about the overall effect of treatments on survivors.

**Table 3:** Effectiveness of Mental Health Interventions by Study

Study	Setting	Mode of Delivery	Type of Intervention and Control (n)	Diagnostic Instrument	Outcomes	Population Group and % that were men
Bolton P et al. 2014a [42]	KRI	Primary health care services, 12 sessions	(i) BADT^a^ (*n* = 114); (ii) CPT^b^ (*n* = 101); (iii) wait-list control (*n* = 66)	HSCL-25, HTQ, Inventory of Traumatic Grief	**BATD**: (i) Depression *d* = 0.60 (all WLC) and *d* = 0.84 (*p* = 0.003) (BATD-controls); (ii) Dysfunction *d* = 0.55 (p < 0.05) (all WLC) and *d* = 0.79(*p* = 0.007) (BATD-controls) **CPT**: (i) Depression *d* = 0.70 (*p* < 0.001) (all WLC) and *d* = 0.44 (CPT-controls) (ii) Dysfunction: *d* = 0.90 (*p* < 0.001) (all WLC) and *d* = 0.63(*p* < 0.05) (CPT-controls)	**Male inclusive** % of BATD that were men: 43% % of CPT that were men: 42% % of all controls that were men: 41%
Bass J et al. 2016 [43]	KRI	Primary healthcare services, 6–12 sessions	Trauma-informed intervention (*n* = 159) vs control (*n* = 50)	HSCL-25, HTQ, Inventory for Traumatic Grief	Depression: *d* = 0.57 (*p* = 0.02) Dysfunction: *d* = 0.53 (*p* = 0.03) Anxiety: *d* = 0.41 (*p* = 0.01) PTSD: *d* = 0.35 (*p* = 0.07) Traumatic grief: *d* = 0.26 (*p* = 0.08)	**Male inclusive** % of intervention that were male: 66% % of controls that were male: 70%
Weiss WM et al. 2015 [44]	Southern Iraq	Primary healthcare services, 8–12 sessions	CETA^c^ (*n* = 98), CPT (*n* = 106), wait-list control (*n* = 109)	HTQ, HSCL-25, Locally developed Function Scale	**CETA**: (i) Trauma: *d* = 2.40, M = -0.59 (all WLC); (ii) Anxiety: *d* = 1.60, M = -0.68 (all WLC); (iii) Depression: *d* = 1.82, M = -0.67 (all WLC); (iv) Dysfunction: *d =* 0.88, M = -0.50 (all WLC) **CPT**: (i) Trauma: *d* = 0.41, M = -0.16 (all WLC); (ii) Anxiety: *d* = 0.27, M = -0.14 (all WLC); (iii) Depression: *d* = 0.40, M = -0.22 (all WLC); (iv) Dysfunction: *d =* 0.07, M = -0.05 (all WLC)	**Male inclusive** % of CETA that were men: 68% % of CETA controls that were men: 72% % of CPT that were men: 67% % of CPT controls that were men: 62%
Bolton et al. 2014b [48]	Thailand	At the client or counselor’s home, local Burmese-run clinics or community organizations, and secluded areas, 7–13 sessions	CETA^c^ (*n* = 148) vs control (*n* = 126)	HSCL-25; HTQ	Depression: M = -0.49 (CI − 0.59,-0.40), *d* = 1.16 (*p* < 0.001) PTS: M = -0.43 (CI − 0.51,-0.35) *d* = 1.19 (p < 0.001) Anxiety: M = -0.48 (CI − 0.61, − 0.34) *d* = 0.79 (*p* < 0.001) Functional impairment: M = -0.44 (CI − 0.59, − 0.28) *d* = 0.63 (*p* < 0.001) Aggression: M = -0.24 (CI − 0.34, − 0.15) *d* = 0.58 (p < 0.001)	**Male inclusive** % of CETA that were men: 39% % of controls that were men: 36%
Bass et al. 2013 [51]	DRC	NGOs, 12 sessions	Group CPT ^b^(*n* = 248) vs Individual psychosocial support (*n* = 157)	HSCL-25, HTQ	Combined depression and anxiety: *d =* 1.8 (*p* < 0.001) at end of treatment; *d =* 1.6 (*p* < 0.001) 6 mos after end of treatment Trauma: *d =* 1.4 (*p* < 0.001) at end of treatment; *d =* 1.3 (*p* < 0.001) 6 mos after end of treatment Functional impairment: *d =* 1.1(p < 0.001) at end of treatment; *d =* 1.2 (*p* < 0.001) 6 mos after end of treatment Probable depression or anxiety: *d =* 7.3 (3.4–16.8) (*p* < 0.001) at end of treatment; *d =* 4.6 (2.1–11.1) (*p* < 0.001) 6 mos after end of treatment Probable PTSD: *d =* 12.3 (5.2–30.5) (*p* < 0.001) at end of treatment; *d = 5.5*(2.5–13.2) (p < 0.001) 6 mos after end of treatment	**Female specific**
O’Callaghan et al. 2013 [62]	DRC	Local secondary school, 15 sessions	Trauma-focused CBT^d^ (*n* = 24) vs. WLC (*n* = 28)	UCLA PTSD Reaction Index; African YPAI	Trauma symptoms: F_1,49_ = 52.708, *p* < 0.001, c_p_^2^ = 0.518 Depression and anxiety: F_1,49_ = 52.371, *p* < 0.001, c_p_^2^ = 0.517 Conduct problems: F_1,49_ = 17.123, *p* < 0.001, c_p_^2^ = 0.259) Prosocial behaviour: F_1,49_ = 5.39, *p* < 0.001, c_p_^2^ = 0.099) At 3 months: PTS symptoms: *d* = 2.04, Depression and anxiety: *d* = 2.45), Conduct problems: *d* = 0.95 Prosocial behaviour: *d* = − 1.57 Depression and anxiety symptoms (4.79 points, 95% CI = 0.617–8.966, *p* < 0.05) and prosocial behaviour (− 3.29 points, 95% CI = -5.046 to − 1.537, p < 0.05) showed continued improvements 3 months after intervention ended	**Adolescent girls**
Hall et al. 2014 [56]	DRC	IRC facilities, 12 sessions	CPT^b^ (n = 157) vs. Individual support (*n* = 248)	HSCL-25, HTQ Integrated Questionnaire for the Measurement of Social Capital	CPT: group membership and participation (*d* = 0.22, *p* < .05;) (compared to IS) Within 1 month: CPT: higher emotional support seeking (*d* = 0.37, *p* < .05;), which was not maintained at 6 months	**Female specific**

^a^ Brief Behavioural Activation Treatment for Depression

^b^ Cognitive Processing Therapy

^c^ Common Elements Treatment Approach

^d^ Cognitive-Based Therapy

There is currently limited evidence on which intervention components are most effective to improve mental health. However, studies with female CRSV survivors suggest that interventions that promote social connectedness, safety, and security can improve mental health [56, 60, 62]. No male-inclusive studies measured the effects of interventions on social connectedness, safety, and security. For female-specific interventions, group therapy or counselling sessions were associated with greater social connectedness and support networks [56]. None of the studies targeted LGBT or sought to identify the sexual orientation or gender identity of the participants.

Hall and colleagues [56] evaluated changes in social capital following group-based CPT for female survivors of sexual violence in DRC. The authors found that participation in group therapy after 1-month follow-up, was associated with a significant increase in emotional support seeking compared to the individual support condition (*p* < 0.05, *d* = 0.37), however this was not maintained at 6-months follow-up. CPT group therapy was also associated with significant improvements in group membership and social participation outside of therapy in comparison to the individual support condition (*p* < 0.05, *d =* 0.22) at 6-months follow-up [56]. In Rwanda, a support group for HIV-positive women was found to increase security among the participants and social connectedness and unity. The support group provided a safe space for participants to share their experiences. This led to a reported decrease in loneliness and isolation, and increased social connection and unity. It also led to greater self-esteem, hope, and self-efficacy and improved physical and mental health as they reported they were more likely to attend medical appointments and engage in social activities [59]. A 15-session Group trauma-focused CBT (TF-CBT) among 12 to 17 year-old female sexual assault survivors in the DRC was found to lead to a significant increase in prosocial behaviours that was sustained 3 months after the intervention had ended [62].

### Theory-building for interventions targeting male and LGBT survivors

#### How gender influences mechanisms of change for health interventions on CRSV

Exposure to violence is associated with high levels of psychological distress and mental health problems [67]. Symptoms of poor mental health among male survivors of sexual violence include poor emotional regulation and anger, alcohol and drug abuse, impaired memory and concentration, depression, anxiety, hopelessness, low self-esteem, difficulty relating to others or engaging in intimate relationships, self-mutilation, suicidal behaviour, sleep disturbances and cognitive impairment [2, 3, 26]. Female survivors manifest many of the same symptoms. However, research suggests that internalising behaviours, such as anxiety and depression, are more common among women, while men tend to display externalising behaviours, such as antisocial behaviour and substance abuse, more often [68–70].

In their realist review of interventions targeting female survivors of CRSV, Spangaro and colleagues [34] identified five mechanisms deemed to underpin effective interventions, from a survivor’s perspective: “there is help for this problem”; “services are acceptable and feasible”; “it is safe to tell”; “we can work together to address this problem”; and “we have our own ways of dealing with this problem”. The first four mechanisms are linked to knowledge of services availability, access to services, disclosure of violence and acceptance and adherence to intervention. The fifth mechanism suggests the importance of culturally adapted local models of care. Importantly, Spangaro et al. [34] found that interventions with multiple components and combined with community engagement tended to have positive outcomes, although the evidence was limited.

The authors identified that the first necessary condition for CRSV interventions’ effectiveness was the recognition by women and girls that “there is help for this problem”. The literature on male and LGBT survivors suggests that the recognition of sexual abuse as a social problem has a different rationale for male and female survivors. Specific gender differences that hinder disclosure among men are related to confusion, guilt or self-blame around their sexuality [71, 72]. Male survivors of CRSV often have specific misconceptions about male sexual violence, which can contribute to their anxiety and increase the barriers to reporting [73].

Male, female and LGBT survivors may not seek help because of fear of retaliation, lack of protection, and concerns about being rejected by family and friends [41, 58]. Stigma around being a survivor of sexual violence can also prevent survivors in general from seeking help [41, 63]. These feelings may be aggravated among male survivors by difficulty in reconciling hegemonic models of masculinity with expressions of vulnerability [70]. Among gay male sexual assault survivors, internalised homophobia may hamper access to care and, at the same time, is associated with symptom severity in both depression and PTSD [74].

Research suggests that community sensitisation and awareness may be a strategy to overcome the stigma and discrimination surrounding survivors of CRSV access to healthcare [41, 45, 46, 49, 50]. However, in our review, we did not identify models of sensitisation and awareness specifically designed for male and LGBT survivors of CRSV. For example, a multi-care package implemented in the DRC recognised survivors’ barriers to access, and relied on “counsellor mothers” to give health talks in the village that provided information on services, awareness on health issues. These activities aimed to motivate survivors of CRSV to seek confidential care. Drama and theatre performance were also used to address issues of access to care, consequences of not seeking services, legal issues and feelings of guilt among survivors. Nonetheless, the study did not mention how the intervention specifically addressed male survivors and their partners, how men, boys or LGBT persons engaged with the “counsellor mothers” and if issues pertinent to sexual violence against men or boys were represented in drama and theatre activities. It was found that all of the survivors that did seek care came from within a 30 Km radius and were either self-referred, referred through a friend, an NGO, or interacted with the counsellor mothers or saw the theatre performance, although this was not disaggregated by gender. The authors did note that male survivors rarely used the programme [45].

Similar strategies to overcome stigma and discrimination associated with sexual violence were used in another multisectoral intervention in the DRC in which community leaders and community core groups were trained to identify survivors, educate them about the services available, provide psychosocial support and make referrals to medical, legal, and socioeconomic services [41]. Using faith-based organisations and local networks and resources, the project was implemented in areas of eastern DRC where SGBV responses were either non-existent, limited, or had limited referral to services. Again, it was not clear how these groups engaged with males (or not) and how beneficial it was to male survivors of CRSV. Moreover, although research suggests that faith-based organisations can be effective in promoting health in areas as diverse as primary prevention, general health maintenance, cardiovascular health and cancer prevention [75], there are still controversies associated with some specific religious agendas that might conflict with core values of the rights-based westernised policy agenda [76, 77]. We identified an advocacy intervention that offered support to survivors through religious discourses on sexual violence. This included identification of biblical narratives with sexual violence [78], but we did not find any evaluation of its effectiveness.

Mooren and colleagues [46] evaluated a mental health programme in Sarajevo and Central Bosnia. To promote the services, local health authorities and a weekly radio programme disseminated information to community members. To generate uptake of services and reduce stigma and discrimination, the ISANGE One Stop Centre (IOSC) in Rwanda disseminated brochures, broadcast two TV spots and three talk show interviews, and printed 900 standard operating procedures (SOPs) (mainly for police officers) on the prevention and handling of SGBV cases [49]. The Police Gender Desk also held an annual national GBV week. Although, the authors did not provide information on whether there were differences in how (if at all) messages were tailored to men or women [46], this initiative is in line with comprehensive recommendations of integrated inter-sectoral interventions [79], involving community, media and police. Additionally, community feedback emphasised the need for continued ongoing publicity to maintain awareness of the programme [49].

Among *female-targeted interventions*, in South Kivu, DRC, the Foundation RamaLevina (FORAL) trained community health workers (CHWs) mobile health clinics to reduce stigma and discrimination, and increase uptake of services provided [50]. The CHWs, as respected members of the community, built relationships with survivors, educated them about the medical and psychosocial services available, and encouraged them to seek such services. The CHWs also helped FORAL staff tailor the education sessions to community concerns. At the same time, to reduce stigma associated with SGBV, health services were open to anyone. As such, these services were provided either within a primary health centre or just outside the centre. The mobile clinic was integrated into existing services with the intention that they would be seen as part of the ongoing health provision. Women and their male partners could access the services on the dates that the mobile clinic was in the village, during a six-hour timeframe. According to the study, CHWs reported that patients were satisfied with the services provided and appreciated the health education sessions and the relationship that FORAL staff built with the community. It was not clear whether the CHWs were referring to satisfaction of service users inclusive of male partners, or to female patients alone. It was not clear either whether male and LGBT survivors accessed the services, as they seemed to have been advertised as universal coverage [50].

As mentioned previously, at the core of the first mechanism identified by Spangaro et al. [34] is the recognition by women and girls that “there is help for this problem”. For men, boys and LGBT groups who recognise the trauma experienced as a problem for which they would like to seek help, they may be then confronted with the lack of available specialised assistance and resources [16]. For example, according to an exploratory study on refugee men and boys’ experiences of sexual violence in the Syria crisis [16], some healthcare providers reported feeling uncomfortable treating male and LGBT survivors because they felt they lacked the capacity to respond to their needs. Providers reported they were unaware about rectal trauma as a possible result of sexual violence, and the majority of SGBV social workers were women and not sensitised on how to respond to male or LGBT survivors. This was reinforced by limited (or no) experience of treating male and LGBT survivors, as few of these survivors seek help. A review of studies on male survivors of child sexual abuse indicates that negative reactions from providers to men’s disclosure of sexual abuse are directly associated with negative effects on health behaviours [80], which may in turn reinforce the perception that help is unavailable or it is not “safe to tell” [34].

Indeed, male and LGBT survivors might not know about existing services or might think they provide care solely for female survivors [2, 16]. In addition, the services provided to male and LGBT survivors may not be tailored to meet their needs. For example, community centres in the Kurdistan Region of Iraq (KRI) and Jordan providing services for men and boys who experienced sexual violence did not consult survivors on how to engage them and, as a result, activities were of little interest to the participants [16]. For LGBT individuals, they may be wary of attending mental health services as they may think that the mental healthcare providers may try to treat their sexual orientation and gender identity as a mental illness [81].

Furthermore, there is some evidence that, in general, men are less likely to seek help from health care providers for issues as diverse as depression, substance misuse, physical disabilities and stressful life events [82, 83]. This tendency to delay seeking help may hinder the effectiveness of post-rape medical interventions, and increase risks linked to externalising behaviour such as antisocial behaviour, substance abuse and suicidal behaviour [84, 85].

In many conflict-affected countries, men and LGBT individuals who experience sexual violence are not protected by national legal frameworks that recognise only female victims of rape [86]. In addition, in countries where consensual same-sex acts are still criminalised - 70 countries as of March 2019 [87] -, survivors often face reprisals when reporting abuse [3]. Many countries including Iraq, Jordan, and Lebanon have laws that require mandatory reporting of cases of sexual violence by healthcare providers to the police and other public authorities [16]. This deters many survivors who do not wish to pursue legal action or who want to avoid public exposure from seeking health services [16]. In this context, it is important to recognise that, as noted by the Interagency Guidelines for Case Management [88], mandatory reporting is not always in the best interest of the survivor as it can conflict with principles of confidentiality and self-determination and may even put the survivor at greater risk of re-victimisation by the perpetrator. These factors are likely to influence the third mechanism proposed by Spangaro et al. [34]: “it is safe to tell.” Men and LGBT individuals will rightly perceive that it is not safe to tell if the results of reporting sexual abuse are legal procedures against them or further abuse. Men and boys may also not feel that it is safe to tell providers who they perceive have negative attitudes about male survivors of sexual violence [28]. Additionally, gender norms may influence preferences for same-sex providers, and they may prefer disclosing to another male instead of a female [89], as focus group discussions with male refugee survivors have suggested [16]. However, there seems to be no universal consensus on this issue [90] and survivors’ preferences are likely to vary according to individual inclinations, cultural norms and legal context. At the same time, men, boys, and LGBT individuals may be reluctant to come forward as victims of sexual violence, as the perpetrators may be community members and known to the family. Other reasons why men and boys may not disclose sexual violence include: not wanting to create problems within the family, potential economic and emotional dependence on the perpetrator, and fear of exclusion [91]. Although much more research was conducted among female survivors of sexual violence, norms that promote family honour and family respect may also be barriers to reporting for male and LGBT survivors [92].

Research suggests that one-stop model of support for female survivors of GBV may be a potential solution to overcome barriers associated with privacy and confidentiality and potentially increase access to justice [93, 94]. Roka and colleagues [45] assessed a medical intervention which provided a full package of care in a designated room (including medications) to ensure that client confidentiality and privacy were protected. It is unclear, however, how effective this strategy was at maintaining confidentiality, how it impacted male patients’ perception of care, and how it influenced their continuity of care [45]. Furthermore, although one-stop interventions may be effective in addressing acute physical health needs and provide immediate care, they may be insufficient to address psychosocial and mental health needs of survivors if not backed up by a specialised referral network.

In relation to the third mechanism proposed by Spangaro et al. [34] “we can work together to address this problem”, gender may also be at the core of behaviour motivations in help-seeking. Principles of psychological treatment - such as, introspection, emotional expressivity and acknowledgement of difficulties - are often in conflict with hegemonic masculinities [28, 95]. Conversely, male coping strategies often include denial of “weakness” and “closing-up” [28, 96], probably linked to norms condoning self-reliance and emotional control [97]. Research suggests that women are more tolerant of the stigma associated with seeking professional help, more likely than men to recognise their personal need for help, and more open to sharing their problems with other people [98]. This unwillingness to seek help seems particularly pronounced among men who experience gender-role conflict - negative consequences of socialised gender roles [99, 100] - and men who stigmatise help-seeking behaviour [101]. The RCTs identified in our review contribute little to shed light on help-seeking behaviours among male survivors, as all treatment and control groups were selected among survivors who already sought help from the services in which the trials were conducted (i.e. no comparison was possible with men who did not seek help in the first place).

The literature on child sexual abuse suggests that men have greater difficulties in coping with sexual abuse and are less successful in resolving the trauma than women. Additionally, they seem more likely to engage in externalising behaviour, including aggression, risky sexual behaviour and suicidal behaviour. Substance abuse is also a common coping mechanism among male trauma survivors [28]. These inadequate coping mechanisms can possibly create a feedback loop between trauma experience, externalising behaviour and further trauma (e.g. CRSV influences substance abuse which results in depression, leading to more substance abuse, which leads to increased severity of depression symptom, etc.) [102]. These coping mechanisms based on self-reliance [97] may also reflect and reinforce for male survivors the fifth mechanism “We have our own ways of dealing with the problem”, as described by Spangaro and colleagues [34], perpetuating the invisibility and silence around sexual violence against men and boys, and potentially feeding the manifestation of antisocial behaviours.

Although this review found no evidence on male survivors involved in group therapy, there is evidence from female-specific interventions illustrating the benefits of group therapy on mental health [43, 53, 55, 59, 60]. Bass and colleagues [51] conducted a controlled trial of group cognitive behaviour therapy in the DRC for survivors of sexual violence, using individual support as the comparison. Their study found that, in comparison to individual support, group therapy participants had significantly greater improvements in PTSD symptoms and combined depression and anxiety symptoms. In fact, the relative risks of displaying depression or anxiety and PTSD diagnostic criteria were significantly higher for the individual support in comparison to group therapy [51]. Allon [55] implemented two types of eye movement desensitisation and reprocessing (EMDR) therapy on female sexual violence survivors in DRC. One was simple EMDR and the other was EMDR Integrative Group Treatment Protocol (EMDR-IGTP). While the patient recalls memories tied to a traumatic event, a therapist applies bilateral stimulation (horizontal eye movements or alternative right-left taps to parts of the body) [55]. For EMDR-IGTP, group participants draw the trauma they experience while they self-apply bilateral stimulation, repeating the practice until they feel they have processed the trauma. The study found that disturbance level significantly decreased in both individual and group therapy arms [55]. Hall and colleagues [56] evaluated the impact of group CPT, in comparison to individual support, on social capital among female survivors in the DRC. Results found that women in group CPT had significant improvements in group membership and participation in comparison to the individual support arm (*p* < 0.05, d = 0.22). There were no differences between group CPT and individual support on non-kin social networks, instrumental support network size, or financial network size. One-month post-intervention, women in the group CPT had significantly higher emotional support seeking compared with those in the individual support arm [56]. One study looked at how facilitated support groups impacted HIV+ Rwandan women to share their lived-experience and how this impacted their mental health [59]. Women in support groups reported feeling safe, and had an increased sense of connection and unity with other group members. They also reported improved social functioning, mental and physical health and greater self-esteem and self-efficacy. There was a decrease in shame and stigma, and increased understanding about the importance of medication and treatment adherence [59]. In a psychosocial group counselling intervention among Afghan female survivors, women stated that through the eight-months of group counselling, their mood and behaviour improved, they learned social skills, family interactions improved, they felt they were able to deal better with stress and make decisions more easily [60]. Support groups may also be effective for men and boys who are able to share their experiences and disclose sexual abuse in a group setting; however, many male survivors may find sharing difficult [103]. An RCT of group versus individual CPT among military personnel seeking help for PTSD (most of whom were male) suggested that individual therapy was associated with greater improvement in PTSD severity when compared to group treatment. At the same time, the effect of group and individual CPT was similar for depression symptoms and suicidal ideation [104]. We did not find any specific studies on the effectiveness of group therapy for male and LGBT survivors of CRSV.

The mechanism “services are acceptable and accessible” will likely depend on whether providers have been sensitised and trained on care for male survivors of CRSV, and whether local norms are in line with the services’ presentation and model of care. Research has suggested that the fear of negative reactions, such as homophobia, transphobia, disbelief, and blame from the police or medical services may prevent male survivors from disclosing sexual abuse and accessing timely services [16, 65, 105]. Indeed, one of the reasons for non-recognition of sexual violence against men and boys in medical, legal and social services [3, 105] seems related to entrenched gender norms, perceptions, beliefs and attitudes of providers. For example, entrenched gender and social norms in the community that promote traditional male roles may also influence the healthcare providers’ response to men, boys, and LGBT survivors. Research suggests that providers may be dismissive, hostile, discriminatory, and not believe survivors [16]. For individuals with diverse sexual orientations and gender identities, accessing supportive and safe services is difficult. Seeking such services can lead to harm, exclusion, and dismissive providers who do not believe the sexually violent act was non-consensual. They often do not have access to services that are sensitive to their needs and may be labelled as not prioritised for assistance [81, 106]. This can lead to a lack of access to and poor quality healthcare [65, 107].

Rape myths that hinder the visibility of sexual violence against men are associated with gender stereotypes, hegemonic masculinities and discrimination of LGBT groups [73, 105, 108]. Survivors and providers often share the belief in these myths. For example, studies have suggested that even workers at rape crisis centres may sometimes share common prejudices about male sexual assault [105]. When comparing providers’ attitudes towards male versus female survivors of sexual violence, research has shown that less sympathy is usually displayed in relation to male survivors. LGBT survivors are also more likely to be blamed than heterosexual survivors, including the perception that “LGBT individuals deserve to be sexually assaulted because they are immoral and deviant” [74, 105]. As a consequence, these negative attitudes are likely to reinforce survivors’ self-blame and hinder recovery [105, 109].

Some common misconceptions and prejudices that can contribute to both underreporting and under-identification of cases include: men cannot be raped; real men can defend themselves against rape; women cannot sexually assault men; men are not affected by rape; male rape only happens in prisons; sexual assault by someone of the same sex causes homosexuality; male rapists and their victims tend to be homosexuals; homosexual and bisexual individuals deserve to be assaulted; and if a victim physically responds to an assault he must have wanted it [28, 74, 105]. These misconceptions are derived from traditional views of masculinity which reinforce strength, assertiveness, sexual dominance and heterosexuality [105].

Nonetheless, our review found that there have been efforts to improve healthcare providers’ knowledge and attitudes about survivors of CRSV through sensitisation, awareness and training [43, 64–66, 110, 111]. A multimedia training tool to improve clinician knowledge, attitudes, and practices about sexual assault survivors was implemented and evaluated by the International Rescue Committee (IRC) in refugee camps in Ethiopia and Kenya, post-conflict setting in DRC, and an urban refugee setting in Jordan [65, 66]. The tool sensitises healthcare providers on the following topics: knowledge about sexual assault, beliefs affecting survivors, and patient rights; non-medical staff responsibilities in engaging with survivors; patient clinical care for survivors; and ensuring the facility has the resources to address survivors’ needs [65]. Pre- and post-intervention results found that female healthcare providers and those who had prior experience working with survivors experienced an increase in positive attitudes pre- and post-intervention. Respecting patient’s rights, including the right to self-determination and the right to non-discrimination, increased post-intervention. Blaming survivors and negative beliefs about sexual assault, however, were common among healthcare providers and did not significantly decrease post-intervention. Yet healthcare providers stated that they could put aside their personal beliefs to ensure that the patient’s rights were respected. Questioning survivors’ credibility about their sexual assault claim was common and did not decrease post-intervention, nor did the belief that sexual violence cannot happen between intimate partners. Clinical care knowledge and confidence improved three months post-intervention. Healthcare providers were more likely to obtain informed consent, employ active listening skills, and give survivors more control over their exam. There was a significant increase in provider’s ability to identify the emotional and physical reactions that male survivors experience. However there was no improvement in their knowledge on adaptations that should be made to the physical exam. There was also a significant increase in provider’s ability to obtain informed assent from children, perform a physical exam, and identify at which age emergency contraception should be offered; however, there was no increase in provider’s knowledge of child survivors HIV Post-Exposure Prophylaxis (PEP) treatment protocol. Although there was an improvement in healthcare providers following clinical care protocols for survivors post-intervention, psychosocial referrals did not improve [65, 66]. There was no report of intervention’s effect disaggregated by gender of survivors, which hinders conclusion about effectiveness of the trainings associated with care provision for male or LGBT survivors, especially considering that previous research has indicated reduced empathy with these groups of survivors [74, 105].

As part of a mental health RCT in Kurdistan region of Iraq (KRI), Bass and colleagues [43] developed a curriculum for healthcare providers, training them on providing therapeutic care to survivors of torture and trauma using a “social work model of helping and support”. Providers were trained to provide empathetic and compassionate care, and active listening and problem solving. The curriculum also included a component on working with survivors to enhance the therapeutic relationship. To ensure that healthcare providers maintained the treatment model, monthly on-site group supervisions by a psychiatrist, weekly check-ins via mobile phone, and medical record reviews took place [43]. In an evaluation of a community-based medical care programme in Burma that sought to train community health workers (CHWs) and traditional birth attendants (TBAs) using the WHO’s 2004 *Clinical Management of Rape Survivors: Developing protocols for use with refugees and internally displaced persons* curriculum [64], the study found that CHWs were comfortable with the topic of GBV and knowledgeable about the clinical skills necessary to treat survivors of sexual assault (including confidentiality, use of forms, and process). CHWs reported that they were not as confident in taking the patient’s history and providing psychosocial care. TBAs reported that they were concerned for their own safety when engaging with survivors, although they would not allow this to deter them from providing care. Data on male survivors was not provided [64].

The review found several ways that interventions can provide accessible services to survivors of sexual violence. Training community leaders and community core groups [41], and training community members [45] to provide information on health and psychosocial care may provide more accessible services and information to survivors of sexual violence that do not necessitate travel to a health facility [45]. Internet-based therapy can be used as a way to provide accessible psychological care to underserved populations, as was the case of Interapy in Iraq [47]. Participants that used Interapy experienced a significant decrease in PTSD, intrusions, avoidance, and hyperarousal and a significant increase in quality of life post- internet-based therapy. However, due to the nature of internet-based therapy, individuals with severe mental health issues could not participate. At the same time, due to the limited medical infrastructure in Iraq, referrals to mental healthcare professionals for further care was not possible [47]. In addition, ensuring that local service organisations are involved and that survivors can relate to counsellors may also be another avenue to ensure that services are accessible to survivors, as was the case among Burmese refugees in Thailand. The study found that Burmese refugees experienced improvement in depression, PTSD, and anxiety [42]. Home visits were also used to ensure that healthcare is accessible to rural and underserved populations [58]. However, the provision of medical care in rural and remote places often does not include specialised services so patients have to be referred to facilities that are not easily accessible [58]. In all of the RCTs identified in our review, it is uncertain if and how psychological treatments were accessible and acceptable to men and boys [42–44, 48].

#### Contextual barriers in access to care

Among men who receive assistance, many do not follow up treatment. There is attrition at each stage of the assistance process [2]. For both men and women, insecurity is an important barrier to treatment access and uptake. This was a recurring theme in the literature [42–45, 48, 49, 58]. In an RCT investigating CETA on comorbid mental health disorders among Burmese refugees in Thailand, Bolton and colleagues [42] reported that participants were lost-to-follow-up due to lack of time, returning to their home country, changing circumstances, and death, while some were not located. In an evaluation of the ISANGE One Stop Centre (IOSC) in Rwanda, which provides a multisectoral package of medical, psychosocial, legal, and police services to survivors of SGBV, follow-up became an issue once survivors returned to their communities. This was attributed to a lack of resources, limited time, and poor local level care which increased survivors risk to further violence [49]. Similar results were found in a female-specific intervention with FORAL staff and the mobile clinic. The mobile clinic was in the village 4 times per month, and approximately 70% of patients returned for one follow-up visit. However, follow-up dropped to 7 and 3% on the second and third visits, respectively [50].

In settings where the nature and duration of the conflict are particularly severe, health systems may be largely affected or non-existent [41, 43–45, 47, 112]. In many settings, the presence of armed groups hinders dislocation from home to the nearest point of care both for clients and providers, and affects home visits. Looting and pillage of health facilities may also reduce adherence by forcing clients to travel further to seek care [44, 45, 63].

Additionally, in the context of humanitarian emergencies, access to and effectiveness of mental health services depends on the basic needs of survivors being addressed. Mental health is unlikely to be prioritised by survivors who are struggling to feed themselves or find shelter [63]. At the same time, mental health can deteriorate if these needs are not met [113]. Factors such as poverty and armed conflict may act as daily stressors in the lives of CRSV survivors, and can further hinder access to basic health services, compromising positive health outcomes [114].

For male survivors, masculine cultural models denote the responsibility of financially supporting their families, which may also affect their psychosocial wellbeing and recovery, especially when access to livelihood options is hindered [16].

#### Gender differences on treatment effectiveness

If all the conditions in the mechanisms described above are met and men decide to “work together to address the problem” [91], there may still be potential gender differences in motivation, commitment and responses to psychological treatment between men and women [115]. Indeed, research has identified persisting gender differences in the prevalence, symptomatology and risk factors of mental health disorders [97, 116, 117]. Our review did not find specific data for male and female adolescents, and LGBT persons in different age ranges, nor did it find specific studies on CRSV.

Although there is evidence for a comparable immediate effect of CBT on men and women [91], an RCT of CBT for PTSD found that gender is a predictor of long-term response to treatment, with women maintaining more gains than men [118]. Similar results were found in a systematic review of gender differences for PTSD interventions, with women more likely to experience a greater decrease in PTSD symptoms in comparison to men [119]. The authors caution, however, on making definitive conclusions on the basis of these comparisons. They state there could be differences in “treatment quality and fidelity, the type of control condition, and the level of general functioning of patients which may help to explain the finding that women appear to respond better to psychological treatments for PTSD” [119, 120]. Cason and colleagues [120] suggest that women may respond better to PTSD treatment because they have been raised to be more emotionally expressive than men; they may rely on more social support through recovery; and they may generate a stronger therapeutic alliance. Also, men are more likely to express anger, which may compete with the expression of fear required for processing the traumatic event [120, 121].

CETA has been considered as a promising therapeutic avenue for low-resource settings because of its flexibility, capacity to manage comorbidity within a single treatment approach, and reduced required training time and human resources [89]. Although RCTs indicate a positive effect to CETA, impact indicators are not disaggregated by gender [122].

Overall, the scarcity of disaggregated data does not allow for definitive conclusions on gender differences in treatment effectiveness by gender, gender identity, or sexual orientation.

#### Male and LGBT survivors of CRSV in health guidelines and protocols

Table 4 presents the guidelines reviewed for the present paper, indicating the author, title, year and edition for each guideline.

**Table 4:** List of guidelines analysed under the rapid review

Author	TitTitle	Year
GBV Area of Responsibility (AoR)	The Inter-Agency Minimum Standards for Gender-Based Violence in Emergencies Programming	2019a
GBV Area of Responsibility (AoR)	Guidelines for Integrating Gender-Based Violence Interventions in Humanitarian Action	2015
GBV Area of Responsibility (AoR)	Handbook for Coordinating GBV in Interventions in Humanitarian Settings	2010
GBV Area of Responsibility (AoR)	Handbook for Coordinating GBV in Interventions in Humanitarian Settings	2019b
Gender-based Violence Information Management System (GBVIMS) Steering Committee	Inter-Agency Gender Based Violence Case Management Guidelines: Providing Care and Case Management Services to Gender-Based Violence Survivors in Humanitarian Settings.	2017
Global Education Cluster et al.	Guidelines for Child Friendly Spaces in Emergencies. Field testing version developed and reviewed by the Global Education Cluster, Global Protection Cluster – Child Protection Area of Responsibility, Inter-agency Network for Education in Emergencies and the IASC	2011
Global Protection Cluster/Child Protection Working Group (Sphere Project)	Minimum Standards for Child Protection in Humanitarian Action	2012
The Alliance for Child Protection in Humanitarian Action	Minimum Standards for Child Protection in Humanitarian Action	2019
Global Women’s Institute, World Bank and Inter-American Development Bank	Violence Against Women and Girls (VAWG) Resource Guide: Health Sector Brief	2015
Global Protection Cluster/Child Protection Working Group (Sphere Project)	Inter-Agency Guidelines for Case Management & Child Protection	2014
IASC (Inter-agency Standing Committee)	Pocket Guide: How to support survivors of gender-based violence when a GBV actor is not available in your area.	2015b
Inter-Agency Standing Committee (IASC) Task Force on Gender and Humanitarian Assistance	Guidelines for Gender-based Violence Interventions in Humanitarian Settings	2005
Inter-Agency Standing Committee (IASC) Sub-Working Group on Gender in Humanitarian Action	Caring for survivors of sexual violence in emergencies. Training guide	2010
Inter-Agency Standing Committee (IASC) Sub-Working Group on Gender and Humanitarian Action	Establishing Gender-based Violence Standard Operating Procedures (SOPs) for multi-sectoral and inter-organisational prevention and response to gender-based violence in humanitarian settings	2008
Inter-Agency Standing Committee (IASC)	Guidelines on Mental Health and Psychosocial Support in Emergency Settings	2007
Inter-Agency Standing Committee (IASC)	Guidelines on Mental Health and Psychosocial Support in Emergency Settings - Checklist for field use	2008
IASC (Inter-agency Standing Committee) Reference Group on Mental Health and Psychosocial Support in Emergency Settings	IASC Reference Group Mental Health and Psychosocial Support Assessment Guide	2012
IASC (Inter-agency Standing Committee) Global Protection Cluster Working Group and IASC Reference Group for Mental Health and Psychosocial Support in Emergency Settings	Mental Health and Psychosocial Support in Emergency Settings: What Should Protection Programme Managers Know?	2010
IASC (Inter-agency Standing Committee) Reference Group on Mental Health and Psychosocial Support in Emergency Settings	Mental Health and Psychosocial Support in Emergency Settings: What Should Humanitarian Health Actors Know?	2010
Inter-Agency Working Group on Reproductive Health in Crises (IAWG)	Inter-agency Field Manual on Reproductive Health in Humanitarian Settings (Revision for Field Review)	2010
Inter-Agency Working Group on Reproductive Health in Crises (IAWG)	Inter-agency Field Manual on Reproductive Health in Humanitarian Settings	2018
International Rescue Committee (IRC), UNICEF	Caring for Child Survivors of Sexual Abuse: Guidelines for health and psychosocial service providers in humanitarian settings	2012
International Rescue Committee (IRC), UNICEF	Advancing the Field: Caring for Child Survivors of Sexual Abuse in Humanitarian Settings (A Review of Promising Practices to Improve Case Management, Psychosocial & Mental Health Interventions, and Clinical Care for Child Survivors of Sexual Abuse)	2011
UNFPA	Minimum Standards for Prevention and Response to Gender-Based Violence in Emergencies	2015
UNFPA, UN Women, WHO, UNDP, UNODC	Essential Services Package for Women and Girls Subject to Violence Core Elements and Quality Guidelines	2015
UNFPA	Managing Gender-based violence programmes in emergencies	2012
UNFPA and Save the Children	Adolescent Sexual and Reproductive Health Toolkit for Humanitarian Settings: A Companion to the Inter-Agency Field Manual on Reproductive Health in Humanitarian Settings (see IAWG entry)	2009
UNFPA	A practical approach to GBV: A programme guide for health care providers and managers	2001
UNHCR	Sexual Violence against Refugees: Guidelines on Prevention and Response	1995
UNHCR	Sexual and Gender-Based Violence against Refugees, Returnees and Internally Displaced Persons: Guidelines for Prevention and Response	2003
UNHCR	UNHCR Handbook for the Protection of Women and Girls	2008
UNHCR	SGBV prevention and response - A training package	2016
UNHCR	Working with Lesbian, Gay, Bisexual, Transgender & Intersex Persons in Forced Displacement	2011
UNHCR and Refugee Law Project (RLP)	Working with Men and Boy Survivors of Sexual and Gender-based Violence in Forced Displacement	2012
UNHCR	Operational Guidance. Mental Health & Psychosocial Support Programming for Refugee Operations	2013
WHO	Guidelines for medico-legal care for victims of sexual violence	2003
WHO	Responding to intimate partner violence and sexual violence against women: WHO clinical and policy guidelines	2013
WHO, UNODC	Strengthening medico-legal responses to sexual violence	2015
WHO	Responding to children and adolescents who have been sexually abused	2017a
WHO	Strengthening Health Systems to Respond to Women Subjected to Intimate Partner Violence or Sexual Violence: A Manual for Health Managers	2017b
WHO, UNFPA, UNHCR	Clinical Management of Rape Survivors - Developing protocols for use with refugees and internally displaced persons	2004
WHO	Health care for women subjected to intimate partner violence or sexual violence: A clinical handbook	2014
WHO	Mental health and psychosocial support for conflict-related sexual violence: principles and interventions	2012
WHO	mhGAP Intervention Guide for mental, neurological and substance use disorders in non-specialized health settings	2010
WHO, UNHCR	mhGAP Humanitarian Intervention Guide (mhGAP-HIG) - Clinical Management of Mental, Neurological and Substance Use Conditions in Humanitarian Emergencies	2015
WHO	mhGAP Intervention Guide for mental, neurological and substance use disorders in non-specialized health settings - Version 2.0	2016
WHO, UNHCR	Assessing mental health and psychosocial needs and resources. Toolkit for humanitarian settings.	2012
WHO	RESPECT women - Preventing violence against women	2019
Jhpiego, U.S. Centers for Disease Control and Prevention (CDC), and WHO	Gender-based violence Quality assurance tool – MINIMUM CARE VERSION	2018

The results of our rapid review of forty-nine international guidelines, protocols and documents guiding policy and practice in the field suggest that evidence about male and LGBT survivors remains limited. Initiatives in the area are increasing nonetheless. Although almost all the documents analysed adopt an inclusive understanding of sexual violence and acknowledge male survivors to varying degrees, the majority of them do not articulate recommendations on how to design and implement interventions that respond to the specific needs and concerns of male and LGBT survivors. In this context, it is important to acknowledge that several principles, procedures and contents underpinning medical and MHPSS care and service provision for female survivors of sexual violence also applies to male and LGBT survivors and that the lack of specific recommendations for these groups does not necessarily equate with lack of guidance. It is also important to highlight that some guidance present in some analysed guidelines – such as GBV guidelines – is related to services and care provision exclusively designated for women and girls including in specifically dedicated spaces such as women and girls’ safe spaces.

However, the review also shows that male and LGBT survivors of sexual violence are increasingly considered in international guidelines and that specific recommendations and guidance is being formulated to manage and respond to cases of sexual violence perpetrated against men, boys and persons who identify as LGBT and better tailor medical and MHPSS services and responses for these groups. One document is entirely focused on male survivors [32] with detailed guidance on how to address their specific needs and vulnerabilities and/or considerations for inclusive sexual violence programming for men, women, girls and boys. One document exclusively focuses on working with LGBT people, including in regard to preventing and responding to sexual violence [123]. Several other guidelines, while keeping the centrality of prevention, mitigation and response to violence against women and girls, call for the need of specific considerations for responding to the needs of male survivors’, including via additional services, diverse and alternative entry points, staff with specialised skills and referral pathways. Some of these documents include specific guidance and resources to support male and LGBT survivors and provide timely access to services that meet their needs [31, 32, 88, 124–127], which represent important developments. This positive trend is also reflected in the increasing consideration that at least three documents have devoted – from one edition to the following – of male and LGBT survivors and highlighted the importance to take their needs, risks and vulnerabilities into account [67, 128–132].

Yet evidence on the implementation, evaluation and effectiveness of these guidelines is sparse. In the literature review, we found two studies that reported on guideline implementation [63, 64]. Mbeya and colleagues [63] reported on International Medical Corps’ implementation of the WHO *Mental Health Gap Action Programme (mhGAP)* as a way to build healthcare provider capacity to respond to those with mental health disorders in CAR [63]. Tanabe and colleagues [64] evaluated a pilot project that used the WHO’s Clinical Management of Rape Survivors to train healthcare providers on community-based medical care for sexual assault survivors in Burma. Another study stated that WHO clinical management of rape protocols were displayed on the walls of the health facilities and disseminated to health facility managers [66] yet it is unclear whether these protocols were implemented by health care providers. No data was available on how they were interpreted and applied in the case of male and LGBT survivors of CRSV.

## Discussion

Our review identified few evaluations that included male survivors of CRSV, and no studies that focussed solely on male or LGBT survivors. Additionally, evaluations that included men did not present results of the analysis disaggregated by gender, sexual orientation, or gender identity and did not explicitly describe components that were designed for men and boys, or the potential implications of interventions for male survivors. To our knowledge, this is the first systematic realist review investigating medical and MHPSS interventions for men, boys, and LGBT survivors of CRSV.

The scarcity of data may partially be associated with the more recent focus in the field on males and LGBT persons experiencing CRSV when compared to women and girls, and the ensuing debates around the implications for resource allocation in the field [1, 3]. Additionally, research regarding LGBT people may be constrained by hostile cultural environments and local punitive legal standards relevant to homosexuality and gender nonconformity [133]. Though some studies acknowledge the existence of sexual minority men, none of the interventions studied targeted LGBT people, and none sought to identify the sexual orientation or gender identity of the participants. The term LGBT itself is probably misleading when describing the study populations in the research field focussing on CRSV. For instance, lesbian, transgender and intersex populations were not mentioned in the studies identified by our review. Also, the term may cause confusion among healthcare providers as LGBT incorporates different groups based on sexual orientation and gender identity. The use of ‘LGBT’ tends to homogenise their experiences as a single social group despite having different vulnerabilities and needs [134]. This may lead to limited awareness about the needs of each of these population subgroups and poor quality healthcare [135].

Humanitarian responses in politically fragile, insecure and resource-limited settings follow political agendas and priorities that depend on the policy timing and its interaction with other policies and local actors [136]. And while communication technology has been recognised as a channel through which conflict-affected communities may articulate their needs and priorities for assistance, the international humanitarian system is still catching up with these potential technological avenues, a delay that is possibly partially caused by existing funding gaps [136].

To date, CRSV against males and LGBT people has remained relatively invisible in humanitarian responses [3, 17, 86, 137]. Survivors often do not disclose abuse, and providers are often unprepared to investigate and respond [3, 16, 138]. Sexual abuse against men, boys and LGBT persons is frequently surrounded by misconceptions and myths that hinder access and provision of care [73]. Interventions that aim to increase self-disclosure and the identification of male survivors will need to address these misconceptions and prejudices about CRSV against men, boys and LGBT survivors. Specifically, future interventions need to rely on the evidence of how self-blame among survivors can be reduced, so that all survivors regardless of their gender identity, “…gender or sexual orientation, can come forward to receive the help that they need without feeling that they will be ridiculed or blamed for their assault” [105].

Mobilisation, sensitisation and capacity building among frontline workers in different sectors can increase entry points for male and LGBT survivors in need of assistance, and can also help reduce invisibility while fostering care for survivors [16, 138]. Human resources that may be well placed to recognise cases, offer referrals and/or assistance may include health practitioners, judiciary and police staff, school staff and teachers, IDP and refugee camp staff, detention centres, and safe houses staff [3, 139]. To foster integrated care for survivors, human resources in these key entry points need to have the knowledge and understanding of specific needs of male and LGBT survivors [105].

At the same time, the relation between gender and sexual abuse is influenced by the cultural context and affect how survivors, communities, and providers perceive and react to the problem. These attitudes and behaviours may have important implications for the acceptability and feasibility of models of care. For instance, local actors may be dismissive of “western” humanitarian norms and practices that inform responses to CRSV by international organisations, hindering acceptance, access and proximity to the populations in need of assistance [136]. The engagement of local authorities, religious leaders, traditional healers and community influencers can inform the design, planning and implementation of interventions [63]. Through sensitisation and awareness, these community resources may help increase referrals and treatment adherence, and reduce the stigma around mental health issues [41, 45, 50, 63]. Non-western therapeutic approaches may also hold some promise for interpreting and recovering from experiences of violence in ways that are grounded in the local cultural context [112], as was also evidenced by the cultural adaptation of CETA among Burmese refugees in Thailand [42].

At the same time, contradictions between religious agendas and health promotion should be taken into account in the advancement and implementation of faith-based models of care [76, 77]. Particularly, controversies around religious treatment of homosexuality may hinder universal care targeting all survivors, and especially the LGBT population. Indeed, Christian, Islamic and Jewish scriptures condemn same-sex sexual behaviour, although some leaders of these three religions challenge traditional interpretations and condemn stigma and discrimination of LGBT [76]. If acted upon, these beliefs represent a clear barrier to care.

Nonetheless, faith-based organisations can provide important support in access and provision of health care [76] as was demonstrated through the Ushindi project in the DRC [41]. Models of care integrating local resources are attractive in low-and-middle-income countries, and especially in humanitarian crisis contexts, where the limited mental health infrastructure, funding, and restricted availability of mental health professionals hinders design and implementation of MHPSS interventions [89]. However, the question of how different faith-based groups promote and deliver health care needs to be addressed before integrating their support into promising models of care. Dilemmas around harmful practices that may facilitate sexual violence could also arise in some contexts [140], as is the case with Bacha Bazi (or dancing boys) in military missions in Afghanistan [141].

Additionally, there are gender differences in the way that men, boys and LGBT people experience, process and express the trauma of sexual violence [15, 28, 70]. Self-blame, guilt, self-doubt and internalised homophobia may prevent male and LGBT survivors from seeking help [15]. Men are also less likely to seek help when it may be met with stigma, is perceived as deviating from masculine norms, and negatively affects their notion of self-concept and level of autonomy [121, 142] leading to health inequalities. When men and boys do seek psychosocial or mental health assistance, they also seem to engage, react and respond differently to women and girls [119–121]. Furthermore, interventions and policies may shape gender relations in conflict-affected settings with both intended and unintended consequences [143]. All these issues have so far been understudied and need to be addressed by future research.

However, current research suggests that, because of these gender differences, mental health interventions benefit from gender-relevant approaches. Specifically, interventions need to incorporate culturally and gender appropriate ways of addressing male survivors’ particular experiences and expressions of trauma and psychological suffering. Among symptoms common to male survivors, externalising behaviours such as anger, aggression and substance abuse deserve some dedicated attention in order to prevent further harm to self and to others [28, 121, 144]. The World Health Organisation [145] also recommends three approaches to address gender inequality issues in treatment access and response. This includes: (i) regulatory approaches, or policies and laws that protect patient and human rights, as well as prohibits discrimination; (ii) organisational approaches that incorporate gender into all facets of the health system, such as budgeting, mainstreaming, assessing and ensuring health outcomes are divided by gender; (iii) informational approaches, or using gender equity indices and health indicators in a country’s health information system [145].

Psychological interventions in conflict settings need to be brief, low-cost, and optimise resources [146]. Primary care may be a promising setting for provision of care to survivors of CRSV. These services can integrate screening and brief interventions to identify and refer survivors to specialised services, prevent mental disorders for those with subthreshold symptoms, increase awareness about mental health and reduce barriers to care. Access may be facilitated because there seem to be less stigma associated with seeking care in primary health facilities as opposed to services solely serving sexual violence cases [113, 147]. Primary health services can also be an effective entry point into the system, especially if local explanatory models and help seeking behaviour are in line with what these type of services’ discourse on health and what they have to offer [147].

Nonetheless, in order to respond effectively to CRSV and survivors’ mental health needs, an inter-sectoral integrated approach is required [148–150]. Mental health and psychosocial support can benefit from integration with access to food and shelter, health, education [132], livelihood, protection and justice [149]. For instance, an RCT measuring the effectiveness of Teaching Recovery Techniques (TRT) delivered by trained counsellors in school settings found significant reductions in post-traumatic stress, depression, traumatic grief, negative school impact, and mental health difficulties in intervention group students compared to the waitlist group [151]. Other examples from our review illustrate the potential benefits of community participation, and media and police collaborations [132]. There are challenges, however, to the implementation of integrated care. The allocation of resources is usually siloed in humanitarian emergencies, and overcoming coordination challenges requires engagement from all sectors involved [113].

In refugee settings, where men, boys and LGBT may be vulnerable to sexual violence [33], there is also the need for health professionals to be sensitised and prepared to address their physical and mental health needs, and link to other sectors to promote protection for survivors. Additionally, the precariousness and instability of life in a camp can also motivate risk behaviours that contribute to poor mental health [152]. Although reports of sexual abuse of women in camps are more widespread, men, boys, and LGBT are also vulnerable to sexual violence, and should have their needs addressed, both in terms of prevention and response. At the same time, care should be taken not to divert attention and resources from the needs of women and girls.

There is also a need to support partners of male and LGBT survivors. Indeed, the “partner’s own grief may severely interfere with any support that the victim may need at this time” - see Coates et al. [153] for a further discussion on negative reactions to rape victims. It must be remembered, however, that partners of male sexual assault victims should not be treated just as an additional support service for the victim, and should be offered treatment in their own right [105].

One promising avenue for intersectoral intervention and service delivery may lie within the technology field. For example, technology is being used to train providers that engage with sexual violence survivors [110, 111]. Physicians for Human Rights (PHR) has developed MediCapt, a mobile phone app that has been developed to link medical, law-enforcement, and legal sectors to facilitate the comprehensive forensic documentation of evidence for survivors of sexual violence. It helps healthcare providers conduct medical exams through the provision of a medical intake form and mobile camera and to securely transmit this data to counterparts in the police and in the legal sectors [110, 111]. It is currently being field-tested. This intervention has not yet been evaluated, and potential effects on identification of cases and health care provision for male and LGBT survivors of CRSV remain unknown.

Several UN agencies and international NGOs have developed guidelines for the prevention and response to survivors of CRSV. In our consultation with experts, there was widespread recognition of the key role and importance of these documents. Our rapid review of forty-nine documents including guidelines, protocols, manuals and other documentation developed by key UN agencies and interagency bodies, showed that despite an increasing consideration of male and LGBT survivors and growing specific recommendations and guidance to better tailor medical and MHPSS services and responses to these groups, only some guidelines include detailed guidance on how to address male and LGBT survivors’ specific needs and vulnerabilities in programming and service provision. Further evidence is therefore needed to ensure specific guidance is provided on how to design and operationalise a survivor-centred, gender-sensitive and intersectional approach to sexual violence programming that addresses the needs of male and LGBT survivors and takes into account sub-groups’ multi-layered vulnerabilities. Future research should also focus on the implementation and effectiveness of these guidelines and collect gender and age disaggregated data.

Research gaps identified in the review included a lack of identification of coping mechanisms used by male and LGBT survivors of CRSV. This could be attributed to lack of gender disaggregated analyses, and that no study focussed on the differing needs of male or LGBT survivors. Similarly, there were no studies that included male or LGBT survivors’ perception and use (or not) of services, and what they consider of value to addressing their needs. This is particularly important as the information could be fed into designing interventions and services tailored to LGBT survivors.

In terms of the quality of medical, mental health and psychosocial care, studies focussed on building healthcare provider competency as a way to improve the quality of care [63–66]. However, the definition of quality healthcare varies from organisation to organisation. For example, according to the World Bank [154], improving the quality of care for survivors of GBV includes not only ensuring competent healthcare providers but also “developing, introducing, and monitoring GBV management protocols and guidelines; screening to ensure early diagnosis and intervention; emotional support & counselling; ensuring privacy, confidentiality and adequate registration; treatment and management of victims of GBV; referral to other services; and community-based care” [154]. On the other hand, according to UNFPA [125], quality psychosocial services are defined as survivor-centred; building resilience at the individual and community level; drawing on family, friends, and community members to support positive coping mechanisms and basic needs; and having access to services [125]. Given the paucity of evidence on what works for male and LGBT survivors of CRSV, as illustrated above with the mention of quality healthcare for survivors of GBV [154], there is limited evidence on what male and LGBT survivors of CRSV deem as quality healthcare. This is an area where future research is needed.

### Limitations

Using a realist approach helped us examine the mechanisms through which medical and MHPSS interventions may work for men, boys and LGBT survivors of CRSV and under which circumstances. There are several limitations, however, to the study. The largest limitation is the lack of data and evidence on male and/or LGBT survivors of CRSV. Studies that included male survivors were not disaggregated by gender, therefore it is unclear how successful the mechanisms of these interventions were in leading to improved health and mental health outcomes for male survivors.

Additionally, the purposive sampling strategy proposed by Pawson and colleagues [37] for theory building in realist reviews does not engage in an exhaustive search of databases, which may lead to partial or incomplete results. Nonetheless, given the exploratory aim of theory building and testing in realist review, the results yielded from this approach are a valuable source of insights and directions for further research and analysis in the field.

Another limitation is that we excluded high-income settings. This is a limitation as higher income settings may have greater evidence on the topic. However, the aim of this paper was to learn about interventions in low-resource settings.

Finally, any effort to systematically review evidence and theory may create an illusion of knowledge completeness among readers that is highly misleading, especially in fields such as social sciences and social epidemiology. Resulting synthesis from such reviews are often only able to reveal a snapshot of what the field has produced in mainstream publication outlets during a given period, and often with important language restrictions. Therefore, their capacity to identify missing perspectives, concepts, evidence and theories is limited. As a result, reviews like ours will necessarily reflect and reproduce some of the biases, limitations, and shortcomings from the mainstream topic area. At the same time that they may not take into account important local definitions of CRSV against different populations, they can hopefully provide an opportunity to highlight these kinds of gap and thus advance future research.

## Conclusion

Our review clearly suggests an evidence gap on health provision to male and LGBT survivors of CRSV. Further research needs to be conducted on male and LGBT survivors of CRSV to inform gender-appropriate and effective responses to the physical and mental health outcomes of these populations. The relatively new focus on research among men who experience sexual violence is not intended to deviate attention, further research, or funding from the pervasive sexual violence that women experience in conflict settings [2, 6, 14]. Instead, it is meant to widen our understanding of how to improve assistance to all survivors, independent of their gender identity or sexual orientation. As noted by Baker and colleagues [155], “any serious effort to improve public health must include attention to the health needs of both sexes and responsiveness to the differences between them”. We agree with the authors and add: to be truly inclusive, these efforts must address the health needs of all individuals of different sexual orientation, gender identity and expression, and sex characteristics.

Gender norms can become embodied in health behaviour and in health provision [156] and perpetuate inequalities for women, girls, men, boys and LGBT persons. Gender-sensitive approaches need to carefully consider and respond to differences in health needs between these diverse groups [157]. However, gender is not the sole aspect of individual and group identities that can increase vulnerabilities to sexual violence, and affect health. Survivors have multiple identities, including ethnicity, religion and political standing that intersect in shaping risks and needs [17].

The almost exclusive focus of the limited existing research and policy on women’s risk of sexual violence obscures the experience of men, boys and LGBT survivors of CRSV [24, 143]. A lack of understanding on how to effectively address the needs of male and LGBT persons may expose these groups to further health and protection risks [158]. It is therefore critical for all health professionals to recognise that the needs of male and LGBT survivors are real and require attention, despite the fact that they are members of a dominant group [28]. In addition, it is important that further research not only disaggregate data by gender but also gender and age. In the literature, girls and boys are often mentioned in conjunction with women and men respectively, but data disaggregated by gender and age is rarely presented.

Similarly, the focus on sexual violence is not meant to detract attention from other forms of violence or GBV that affect men, boys and LGBT persons in conflict settings or in new host communities such as executions, kidnappings, starvation, enforced disappearances, domestic violence, harassment based on gender, forced and early labour and homophobic violence [24]. On the contrary, this focus intends to inform health care models to help create services that address the needs of all survivors.

## Abbreviations

ASP: All Survivors Project
BADT: Brief Behavioural Activation Treatment for Depression
CAR: Central African Republic
CBT: Cognitive-Based Therapy
CETA: Common Elements Treatment Approach
CHW: Community Health Worker
CMR: Clinical Management of rape
CPT: Cognitive Processing Therapy
CRSV: Conflict-Related Sexual Violence
DRC: Democratic Republic of Congo
EMDR: Eye Movement Sensitisation and Reprocessing
EMDR-IGTP: EMDR Integrative Group Treatment Protocol
GBV: Gender-Based Violence
IOM: International Organization for Migration
IRC: International Rescue Committee
KRI: Kurdistan Region of Iraq
LGBT: Lesbian, Gay, Bisexual, Transgender and other gender non-binary Individuals
MHPSS: Mental Health and Psychosocial Support
MSF: Médecins Sans Frontières (MSF)
NGO: Non Governmental Organisations
PEP: Post Exposure Prophylaxis
PTSD: Post Traumatic Stress Disorder
RCT: Randomised Controlled Trial
SGBV: Sexual and Gender-Based Violence
SGM: Sexual and gender minorities
TRT: Teaching Recovery Techniques
UNFPA: United Nations Population Fund
UNHCR: United Nations High Commissioner for Refugees
UNICEF: United Nations Children’s Fund
WHO: World Health Organization
